# Effect of urbanization on bone mineral density: A Thai epidemiological study

**DOI:** 10.1186/1471-2474-6-5

**Published:** 2005-02-04

**Authors:** Chatlert Pongchaiyakul, Tuan V Nguyen, Vongsvat Kosulwat, Nipa Rojroongwasinkul, Somsri Charoenkiatkul, Rajata Rajatanavin

**Affiliations:** 1 Division of Endocrinology, Department of Medicine, Faculty of Medicine, Khon Kaen University, Thailand; 2 Bone and Mineral Research Program, Garvan Institute of Medical Research, Sydney, Australia; 3 Institute of Nutrition, Salaya Campus, Mahidol University, Thailand; 4 Division of Endocrinology, Department of Medicine, Ramathibodi Hospital, Mahidol University, Thailand

## Abstract

**Background:**

The incidence of fractures in rural populations is lower than in urban populations, although the reason for this difference is unclear. This cross-sectional study was designed to examine the difference in bone mineral density (BMD), a primary predictor of fracture risk, between urban and rural Thai populations.

**Methods:**

Femoral neck and lumbar spine BMD was measured by dual-energy X-ray absorptiometry (GE Lunar, Madison, WI) in 411 urban and 436 rural subjects (340 men and 507 women), aged between 20 and 84 years. Body mass index (BMI) was calculated from weight and height.

**Results:**

After adjusting for age and body weight in an analysis of covariance model, femoral neck BMD in rural men and women was significantly higher than those in urban men and women (*P *< 0.001), but the difference was not observed at the lumbar spine. After stratifying by sex, age group, and BMI category, the urban-rural difference in femoral neck BMD became more pronounced in men and women aged <50 years and with BMI ≥ 25 kg/m^2^.

**Conclusions:**

These data suggest that femoral neck BMD in rural men and women was higher than their counterparts in urban areas. This difference could potentially explain part of the urban-rural difference in fracture incidence.

## Background

Osteoporosis and its ultimate consequence of low traumatic fracture pose a major public health problem, because it incurs significant costs and increased risk of mortality [1-3]. Osteoporosis is sometimes considered a "consequence" of modernization, because the incidence of fractures in urban regions is often higher than in rural regions [4-10], although the underlying reason for this trend is largely unknown.

Measurement of bone mineral density (BMD) is considered the primary predictor of fracture risk [11]. Therefore, it could be hypothesized that the urban-rural difference in fracture incidence is partly explained by the urban-rural difference in BMD. However, such a difference has not been well documented due to limited data available [12-14]. Some previous studies reported that rural subjects had higher BMD or bone mineral content (BMC) than those urban subjects [12,13], but another study found no such difference [14].

The pace of urbanization in developing countries is more pronounced than in developed countries. Therefore, developing countries are ideal settings for studying the urban-rural difference in BMD. The aim of this study was to examine the difference in BMD between an urban population and a rural population in Thailand.

## Methods

### Setting and subjects

The present study was designed as a cross-sectional, population-based investigation. The setting was Bangkok city and Khon Kaen province in Thailand. Bangkok is the capital city with a population of 5.7 million and Khon Kaen is a rural province, located 445 km northeast of Bangkok with a population of 1.8 million and is largely an agricultural community. Further details of this study have been described elsewhere [15]

The study included 872 Thai men and women, aged between 20 and 84 years, of whom 422 subjects were from Bangkok and 450 subjects from Khon Kaen. In Khon Kaen, subjects were recruited from 2 villages in the Muang district. There were 14 hamlets in the two villages. In each hamlet, a full list of subjects was obtained, from which 40 subjects were randomly selected by the village's administrator. The selected subjects were then sent a letter of invitation to participating in the study. The response rate was 80.3%. In Bangkok, subjects were recruited via a media campaign, and the sampling technique was similar to the scheme used in Khon Kaen, where subjects were randomly selected from 5 districts within the city of Bangkok.

All Khon Kaen subjects were farmers, while Bangkok subjects were office workers, factory workers or house workers. Twenty-one subjects were excluded from analysis because of bone disorders, chronic diseases, history of taking medications that are deemed to affect calcium and bone metabolism, such as the use of steroids or thyroid hormone; and 4 women were excluded on the basis of pregnancy, lactation, delivery or abortion within the previous 3 months, previous history of oophorectomy and premature menopause. The study was conducted in accordance with the Helsinski Declaration in 1975 and as revised in 1983 and was approved by the Ethics Committee of Faculty of Medicine Ramathibodi Hospital Mahidol University (Bangkok) and Khon Kaen University (Khon Kaen), and written informed consent was obtained from all subjects.

### Measurements

BMD at the femoral neck and lumbar spine (L2-4) in g/cm^2^, was measured by dual-energy X-ray absorptiometry with a Lunar DXP-IQ densitometer (GE Lunar Radiation Corp, Madison, WI, USA). The two study sites (Bangkok and Khon Kaen) used the same model of the DXA machine and the same protocol of measurements. The radiation dose with this method is < 0.1 *μ*Gy. The coefficient of variation of BMD for normal subjects is 0.96 and 0.98 at proximal femur and lumbar spine, respectively.

Body weight (including light indoor clothing) was measured using an electronic balance (accuracy 0.1 kg) and standing height (without shoes) with a standiometer (nearest 0.1 cm). Body mass index (BMI) was calculated as ratio of weight (in kg) over height (in meter squared).

### Statistical analyses

Descriptive statistics were computed for each residential region and sex separately. In order to test for difference between urban and rural regions, an analysis of covariance (ANCOVA) model was performed. In this model, BMD was treated as outcome variables; age and weight (or BMI) were treated as covariates; and residence (urban or rural) was the factor. Interactions between age and BMI or age and residence variable were also considered in the model. Estimates of the model parameters were based on the least square method via the SPSS version 9.0 (SPSS, Inc, Chicago).

## Results

### Demographic characteristics

After excluding 25 subjects, data from 847 subjects (340 men and 507 women) were analysed. There was no significant difference between urban and rural subjects with respect to age or sex distribution. The mean age was 49 and 50 years old in men and women, respectively. However, urban men had higher weight and greater height than rural men (*P *< 0.001), whereas urban women had a greater height (*P *< 0.001) but equivalent weight (*P *= 0.72) compared with rural women (Table 1).

**Table 1:** Characteristics of study subjects

	Urban (Bangkok)	Rural (Khon Kean)	Mean Difference (95% CI)	*P *value
**Men**				
Number of Subjects	159	181		
Age (years)	49.6 ± 17.5	49.1 ± 17.1	0.5 (-3.2, 4.2)	0.800
Body weight (kg)	64.3 ± 11.1	58.2 ± 8.8	6.1 (3.0, 8.1)	<0.001
Height (cm)	165.5 ± 6.3	161.2 ± 5.9	4.3 (3.0, 5.6)	<0.001
Body Mass Index (kg/m^2^)	23.4 ± 3.6	22.4 ± 2.8	1.0 (0.3, 1.7)	0.003
Bone Mineral Density (g/cm^2^)				
Femoral neck	0.87 ± 0.16	0.96 ± 0.18	-0.09 (-0.13, 0.05)	<0.001
Lumbar spine	1.12 ± 0.17	1.11 ± 0.16	0.01 (-0.03, 0.04)	0.64
**Women**				
Number of Subjects	252	255		
Age (years)	50.4 ± 15.1	50.6 ± 15.9	-0.2 (-2.9, 2.4)	0.853
Body weight (kg)	55.5 ± 8.9	55.9 ± 10.5	-0.4 (-2.0, 1.4)	0.718
Height (cm)	154.7 ± 5.4	152.1 ± 5.2	2.6 (1.6, 3.5)	<0.001
Body Mass Index (kg/m^2^)	23.2 ± 3.8	24.1 ± 4.0	-0.9 (-1.5, -0.1)	0.017
Bone Mineral Density (g/cm^2^)				
Femoral neck	0.79 ± 0.13	0.87 ± 0.19	-0.08 (-0.11, -0.05)	<0.001
Lumbar spine	1.05 ± 0.18	1.01 ± 0.21	0.04 (-0.11, 0.08)	0.16

All values are shown in mean ± standard deviation (SD).

In the entire sample, higher weight was associated with higher BMD in men (*r *= 0.13, *P *= 0.017 for femoral neck, and *r *= 0.37, *P *< 0.001 for lumbar spine) and in women (*r *= 0.33, *P *< 0.001 for femoral neck, and *r *= 0.33, *P *< 0.001 for lumbar spine). On the other hand, advancing age was associated with a significant reduced BMD in men (*r *= -0.53, *P *< 0.001 for femoral neck, and *r *= -0.15, *P *= 0.007 for lumbar spine) and women (*r *= -0.63, *P *< 0.001 for femoral neck, and *r *= -0.60, *P *< 0.001 for lumbar spine).

However, the strength of relationship between age and BMD in urban subjects was less pronounced than in rural subjects, such that rural women had a higher cross-sectional "rate of bone loss" than urban women, particularly at the femoral neck. For example, in women, each 5-year increase in age was estimated to associate with a 2.1% and 1.2% decrease in femoral neck BMD for rural and urban group, respectively; in men, the respective rate of decrease was 1.3% and 0.8%. As a result, among those aged 50^+ ^years, BMD in rural subjects tended to be lower than (or converged to) BMD in urban subjects (Figure).

**Figure 1:**
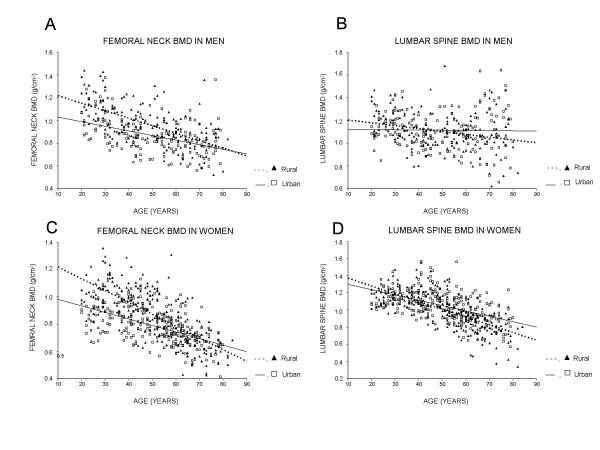
Interaction effects of age and residence variable on bone mineral density at the femoral neck in men (A) and women (C), and at the lumbar spine in men (B) and women (D).

### Urban-rural difference in BMD

In both sexes, after adjusting for age and weight, BMD in rural individuals was significantly higher than in urban individuals. For instance, femoral neck BMD in rural men and women was 0.22 and 0.23 g/cm^2 ^significantly higher (*P *< 0.001) than in urban men and women, respectively; but the difference was lower for the lumbar spine BMD (0.12 g/cm^2 ^in men, *P *= 0.017 and 0.05 g/cm^2 ^in women, *P *= 0.293). The statistical significance of the age-by-residence interaction term in the ANCOVA model suggested that the urban-rural difference in BMD decreased with advancing age (Table 2).

**Table 2:** Effects of age, weight and residence on bone mineral density: estimates of parameters of the analysis of covariance stratified by sex and BMD site

Effect	Estimate ± SE	*P *value
**Men**		
*Femoral neck BMD*		
Age (+5 yr)	-0.020 ± 0.003	<0.001
Weight (+5 kg)	0.015 ± 0.004	<0.001
Residence (Rural)	0.222 ± 0.046	<0.001
Age × Residence (Rural)	-0.012 ± 0.004	0.008
*Lumbar spine BMD*		
Age (+5 yr)	-0.001 ± 0.003	0.874
Weight (+5 kg)	0.030 ± 0.004	<0.001
Residence (Rural)	0.122 ± 0.051	0.017
Age × Residence (Rural)	-0.010 ± 0.004	0.048
**Women**		
*Femoral neck BMD*		
Age (+5 yr)	-0.026 ± 0.002	<0.001
Weight (+5 kg)	0.026 ± 0.003	<0.001
Residence (Rural)	0.233 ± 0.034	<0.001
Age × Residence (Rural)	-0.015 ± 0.003	<0.001
*Lumbar spine BMD*		
Age (+5 yr)	-0.033 ± 0.003	<0.001
Weight (+5 kg)	0.031 ± 0.003	<0.001
Residence (Rural)	0.047 ± 0.045	0.293
Age × Residence (Rural)	-0.009 ± 0.004	0.036

SE, Standard Error.

Notes: Since height was not a significant factor in the analysis of covariance model, it was removed from the final model with no significant change of the results.

Further analyses stratified by sex, age group, and BMI category indicated that the urban-rural difference in femoral neck BMD was more pronounced in the younger age group (< 50 years old) and higher BMI (≥ 25 kg/m^2^). This trend was consistent for men and women. However, for lumbar spine BMD, no significant urban-rural difference was observed in most subgroups, with the exception of women aged ≥ 50 years and BMI < 25 kg/m^2 ^in whom BMD was lower in the rural group compared to the urban group (Table 3).

**Table 3:** Bone mineral density in urban and rural men and women by age group and body mass index

**(a) Lumbar spine BMD**					
Sex	Age (years)	BMI (kg/m^2^)	Bone mineral density (g/cm^2^)		
			
			Urban	Rural	Mean difference and 95% CI

Men	< 50	< 25	0.94 ± 0.16	1.05 ± 0.16	**-0.11^a ^(-0.16, -0.06)**
		≥ 25	0.89 ± 0.12	1.04 ± 0.17	**-0.15^b ^(-0.26, -0.03)**
	≥ 50	<25	0.80 ± 0.15	0.86 ± 0.15	**-0.06^b ^(-0.12, -0.01)**
		≥ 25	0.83 ± 0.11	0.92 ± 0.15	**-0.09^b ^(-0.17, -0.01)**

Women	< 50	< 25	0.85 ± 0.11	0.97 ± 0.13	**-0.12^a ^(-0.16, -0.09)**
		≥ 25	0.92 ± 0.11	1.04 ± 0.13	**-0.12^b ^(-0.20, -0.05)**
	≥ 50	<25	0.70 ± 0.11	0.71 ± 0.15	-0.01 (-0.05, 0.03)
		≥ 25	0.76 ± 0.11	0.82 ± 0.15	**-0.06^b ^(-0.11, -0.01)**

**(b) Femoral neck BMD**					

Sex	Age (years)	BMI (kg/m^2^)	Bone mineral density (g/cm^2^)		
			
			Urban	Rural	Mean difference and 95% CI

Men	< 50	< 25	1.12 ± 0.14	1.13 ± 0.15	-0.01 (-0.06, 0.04)
		≥ 25	1.11 ± 0.09	1.14 ± 0.16	-0.03 (-0.13, 0.08)
	≥ 50	< 25	1.05 ± 0.18	1.06 ± 0.16	-0.01 (-0.07, 0.05)
		≥ 25	1.21 ± 0.19	1.19 ± 0.20	0.02 (-0.11, 0.14)

Women	< 50	< 25	1.14 ± 0.13	1.13 ± 0.14	0.01 (-0.04, 0.04)
		≥ 25	1.21 ± 0.15	1.15 ± 0.13	0.06 (-0.02, 0.15)
	≥ 50	< 25	0.92 ± 0.14	0.83 ± 0.19	**0.09^b ^(0.03, 0.14)**
		≥ 25	1.02 ± 0.17	0.97 ± 0.21	0.05 (-0.01, 0.13)

Statistical significance at ^a^*P *< 0.001 and ^b^*P *< 0.05. Statistical significance is indicated by bold-faced letters.

## Discussion

Osteoporosis has emerged as one of the most common diseases in the aged population, and represents one of the most significant public health problems in Asia [2,16,17]. A consistent trend in osteoporosis is that the incidence of fracture is higher in developed countries than in developing countries; and in any country, the incidence is higher in urban than in rural communities [5-11]. While many factors are hypothesized to be responsible for this trend, BMD is thought to be a primary determinant, because it is the most consistent and robust predictor of fracture risk [1,11].

In the present population-based study, we have shown that BMD in a rural Thai population was significantly higher than in urban population, particularly at femoral neck. The magnitude of difference was more than 1 standard deviation which is clinically relevant. It is difficult to compare the present study's results to previous studies' due to differences in methodology and study design. For instance, Sundberg et al [12] reported that lumbar spine BMD (measured by DXA) in rural adolescents was significantly higher than that in urban adolescents, but there was no significant difference in femoral neck BMD. Furthermore, a study from Southern Sweden suggested that bone mass at the forearm (measured by single-photon absorptiomety) in rural population was significantly higher than in urban population and the difference was more pronounced when comparing a true urban population who had lived their entire life in a city with a true rural population who had never lived in a city [13]. A study from Eastern Poland found that the mean lumbar spine BMD values in every age range were higher in rural population than in urban population, but the difference was not statistically significant [14]. Taken together, these results including ours, suggest that rural subjects tend to have higher BMD than in urban subjects.

The present study's data and design can not elucidate any underlying factors that are responsible for the difference but some propositions could be put forward. The urban-rural difference in femoral neck BMD could be due to the difference in the peak of bone mass levels. In this study, both rural men and women aged between 20 and 30 years had significantly higher BMD than urban counterparts. For example, young rural men and women had significantly higher than urban subjects (1.17 *vs. *1.03 g/cm^2^, [95% CI: 0.07–0.22] in men and 1.02 *vs. *0.86 g/cm^2^, [95% CI: 0.10–0.22] in women). This finding was partially consistent with a previous study [12] and could be explain the fact that the urban-rural difference was mainly found in younger age groups.

This study also found that the urban-rural difference in femoral neck BMD decreased with advancing age. The difference may be attributed to the difference in physical activity between the two populations. Rural populations were generally more physically active than urban populations [18,19]. The rural population in this study was mainly farmers who spend most of their time in rice field long hours of physical activity.

However, the difference was sex- and site- dependent. The difference in femoral neck BMD was much more pronounced than that in lumbar spine and this was more transparent before the age of 50 in men and before the menopause in women. After this age the difference was no longer significant. The data suggested that the rate of bone loss in rural population may be more rapid than in urban population. However, this finding was not consistent with a previous study which demonstrated that the rate of bone loss was higher in urban population compared with rural population [13]. The reason(s) for the higher rate of bone loss in rural population in this study is unknown, but low dietary calcium intake could be a contributory factor [20-22].

The present findings must be interpreted in the context of a number of potential strengths and weaknesses. The data were obtained from a large and well-defined rural *vs. *urban area, which allowed the rural and urban difference to be reliably delineated. The study subjects were Thai, among whom, cultural backgrounds and environmental living conditions are different from Western populations. Thus care should be taken when extrapolating these results to other populations.

## Conclusions

These data have demonstrated that femoral neck BMD in rural men and women was higher than their counterparts in urban areas. This difference could potentially explain part of the urban-rural difference in fracture incidence.

## List of abbreviations

All abbreviations are defined in the text.

## Competing interests

The author(s) declare that they have no competing interests.

## Authors' contributions

Chatlert Pongchaiyakul had an active role in the conduct of this study, obtained and analysed data, and drafted the manuscript. Tuan V Nguyen was involved in the conceptual discussion of this study, and had an active role in data analysis, drafting of the manuscript. Vongsvat Kosulwat, Nipa Rojroongwasinkul, and Somsri Charoenkiatkul had an active role in the study design, and was involved in the conceptual discussion. Rajata Rajatanavin conducted and established this study. All authors contributed to the last version of the manuscript.

## Pre-publication history

The pre-publication history for this paper can be accessed here:


